# Relevance to the higher order structure may govern auditory statistical learning in neonates

**DOI:** 10.1038/s41598-022-09994-0

**Published:** 2022-04-07

**Authors:** Juanita Todd, Gábor P. Háden, István Winkler

**Affiliations:** 1 School of Psychological Sciences, University of Newcastle, Callaghan, Australia; 2 Institute of Cognitive Neuroscience and Psychology, Research Centre for Natural Sciences, Budapest, Hungary; 3 Department of Telecommunications and Media Informatics, Faculty of Electrical Engineering and Informatics, Budapest University of Technology and Economics, Budapest, 1117 Hungary

**Keywords:** Psychology, Neuroscience, Auditory system, Cognitive neuroscience, Sensory processing

## Abstract

Hearing is one of the earliest senses to develop and is quite mature by birth. Contemporary theories assume that regularities in sound are exploited by the brain to create internal models of the environment. Through statistical learning, internal models extrapolate from patterns to predictions about subsequent experience. In adults, altered brain responses to sound enable us to infer the existence and properties of these models. In this study, brain potentials were used to determine whether newborns exhibit context-dependent modulations of a brain response that can be used to infer the existence and properties of internal models. Results are indicative of significant context-dependence in the responsivity to sound in newborns. When common and rare sounds continue in stable probabilities over a very long period, neonates respond to all sounds equivalently (no differentiation). However, when the same common and rare sounds at the same probabilities alternate over time, the neonate responses show clear differentiations. The context-dependence is consistent with the possibility that the neonate brain produces more precise internal models that discriminate between contexts when there is an emergent structure to be discovered but appears to adopt broader models when discrimination delivers little or no additional information about the environment.

## Introduction

Hearing is one of the earliest senses to mature with the developing fetus being capable of registering sound from as early as 18 weeks gestation^1^. By the time a new life exits the mother, it has quite a lot of experience with sound information^2^. We know newborns can recognize familiar voices from birth or earlier^3,4^, localize sound sources^5^, and even separate concurrent sound sources^6^. However, less is known about how sound is processed in relation to its context in the early stages of brain development. This study was designed to further our knowledge on how the neonate brain utilizes sound information within structured sequences.

The recording of auditory event-related brain potentials in this study utilizes a methodology that can be applied in neonates in the same way as in adults^7^. Electrodes are placed over the scalp surface while a structured sequence of sound is presented. This methodology has provided insight into statistical learning in the neonates that enables extraction of the kinds of abstract pattern learning required for music and language learning^8–10^. For sound sequences of predictable structure, with increased exposure, the brain becomes less responsive to predictable events and more responsive to events that mismatch one’s predictions^11^. This is reflected in the event-related potential (ERP) responses. The ability to discriminate between responses to predictable and prediction-violating sounds can therefore be used to infer the existence of internal models of the environment representing a prediction or inference about the sensory input most likely to be encountered next^11–13^. This methodology is ideal for use in neonates because the process occurs automatically and does not require a task or explicit attention^6,7,14–21^.

In adults, the amplitude of the response to sounds that violate established patterns is proposed to reflect a precision-weighted signal influenced by the goodness of evidence upon which the internal model is built (e.g., the stability and reliability of the patterning^22,23^). A significant literature places the precision-weighting in the gain on output from specific cell populations in responsive cortical areas (namely the superficial pyramidal cells) acknowledging that this reflects a hierarchical network interaction both within and between brain regions. In other words, a seemingly simple deviance-detection indicator is subserved by a sophisticated iterative inferential process that operates over many different timescales^23–27^. Within scalp-recorded responses, this process has been linked to change over time in several components of the auditory ERP^28^. In adults, the key difference between pattern-matching and mismatching sound emerges within 100–250 ms after the point of deviance and is maximally recorded over fronto-central electrode sites when a nose or mastoid reference is used. This “mismatch” sensitivity (mismatch negativity or MMN) is captured in a difference waveform but in the tone responses it often overlaps early components known as the N1 and P2 and is considered part of the N2 complex^29–31^. The precision-weighting can be seen both as changes in the response to pattern matching sounds that become less negative with repetition and pattern stability over time and in the mismatch response which becomes more negative over this period.

While the morphology of ERPs is rapidly changing within the first year of life and the stable componentry of adulthood only emerges during childhood, ERP signs of detecting auditory deviance are present right from birth^32^. The neonatal layout of the deviance-related ERP response (derived by subtracting the response to regular sounds from irregular ones) is typically dominated by a positive waveform appearing 200–400 ms from the onset of the deviation (termed the mismatch response; MMR^33^), which may be surrounded by an early and a late negative difference, depending on the acoustic properties of the stimuli, the amount and type of separation between the regular and irregular sounds, etc^34^. Although initially understood to be an indicator of probability^29,30^, the mismatch process shows sensitivity to violations of established sound transitions^27,35,36^ requiring a more contextualized form of learning. This type of statistical learning involves extraction of regularities in how features and objects co-occur in the environment over space and time. Importantly, previous studies showed that properties of the infantile electric brain response to auditory deviance suggest that it reflects a prediction error^37^, similarly to the analogue response in adults^38^. The infantile MMR response is elicited both by violating regularities based on individual sounds and on short sound patterns^39^, bringing up the possibility that, again similarly to adults^40^, they may also reflect the large-scale structure of the auditory context.

The study was designed to determine whether the differential precision-weighting based on large scale structure seen in adults^41–44^ might also be observed in neonates. Here we provide evidence that the brain of newborn infants can indeed form internal models that differentiate sounds based on probability, and weight that response based on precision, but that the propensity to do this depends on contextual factors that may be related to a determination of the inferred value of tracking information over time. Differential responses to sound may only occur when a sound provides information about longer-term structures in the environment. Thus, the same acoustic differentiation may or may not be automatically made, depending on its relevance for revealing important information about the context.

## Results

**Figure 1:**
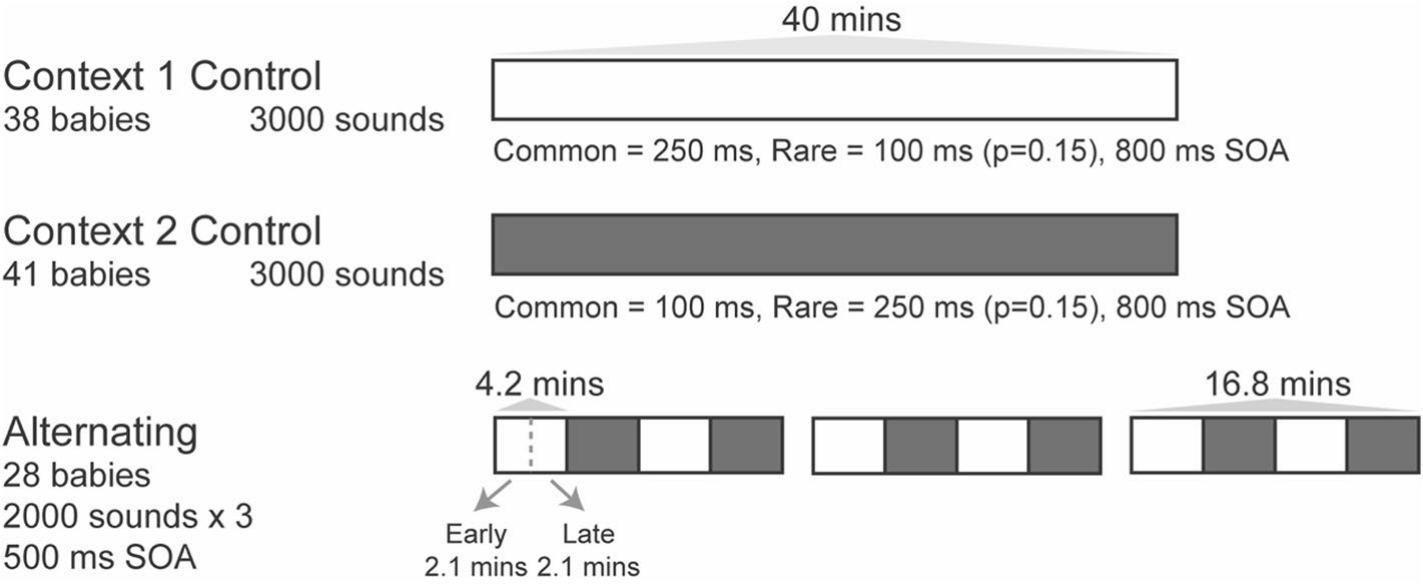
Depiction of the sound sequences used for the control and alternating experiments. The control context sequence was a single stream of sounds about 40 min in length. The alternating sequence contained four × 4.2 min blocks of 500 sounds (16.8 min total length) and it was presented three times with breaks between. For data analysis, the alternating sequence data was further divided to capture early and late periods of time within the two context blocks with indicative periods marked in the figure.

The study featured three experiments with three separate groups of neonates with the sound sequence design depicted pictorially in Fig. 1. In the first two experiments, the sequences heard by babies contained just two sounds; a long tone (250 ms) and a shorter tone (100 ms) that both comprised an overlay of 500 Hz, 1000 Hz and 1500 Hz frequencies. The sounds were organized as traditional “oddball” sequences^11^ such that the two sounds were delivered with largely different probabilities (long p= 0.85 and short p= 0.15, respectively). Experiment 1 is referred to hereafter as the context 1 control. A different group of newborns heard similar sequences but with the sounds in reverse probabilities (Experiment 2), which is hereafter referred to as the context 2 control. When adults hear sequences of this kind, the responses to the common and rare tones differentiate such that the response becomes more “suppressed” or more positive at fronto-central scalp sites with increased exposure, and “larger” or more negative to rare deviations^11^. Stable sequences of this kind are also considered ideal for formation of precise internal models and were designed based on sequences for which probability-dependent response were observed previously in neonates^16^. However, neither group of infants produced responses to rare tones that were significantly different to the common tones, and indeed the responses from each group were remarkably similar, both in amplitude and morphology.

**Figure 2:**
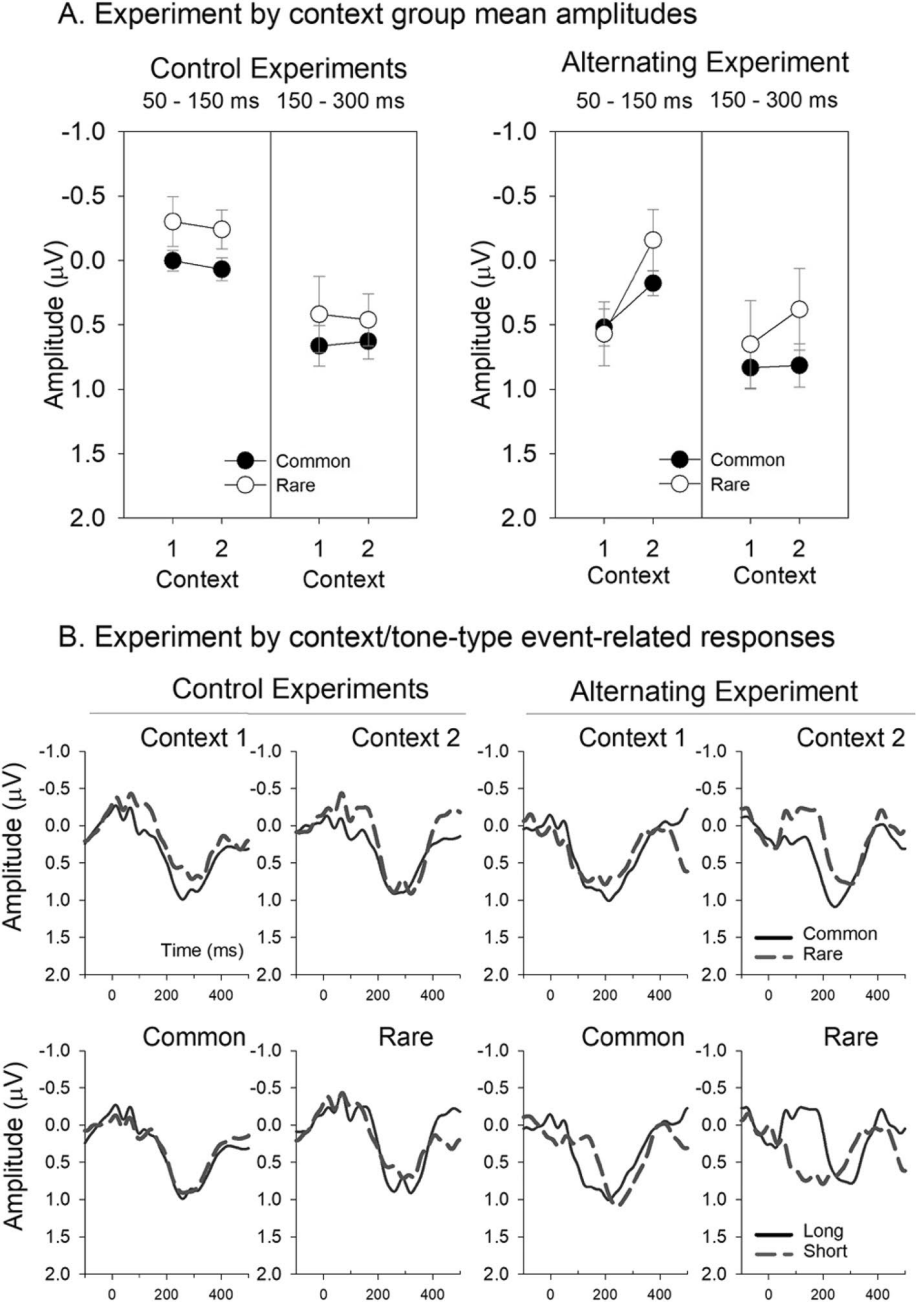
(**A**) Group mean amplitudes over 50–150 ms and 150–300 ms from tone onset for the common and rare tone responses in the two control experiments (left) and the alternating experiment (right), separately for the two contexts. The error bars represent the standard error of the mean. (**B**) Group averaged event-related brain responses at Cz for the common and rare tones in the two control experiments (left plots) and the alternating (right plots) experiment. Event-related brain responses are presented in two ways: (I) overlaid tone type by context (top) and (II) overlaid tone duration by probability (bottom).

In auditory ERPs, the early period of response is considered to reflect relatively more sensory-based processes with the later components progressively more cognitive in nature^45^. In newborn infants, the latency of responses elicited by auditory sensory deviance have been found to depend on both the specific acoustic feature and the amount of separation^32^. In previous neonate studies of tone duration sensitivity, responses to common and rare sounds tended to differentiate at about 150 ms through to 300 ms^16^. To capture this window, responses were quantified using the mean amplitude over 150–300 ms at the Cz scalp electrode. An additional period of analysis was included to capture earlier components using a mean amplitude over 50–150 ms. The component amplitudes are presented in Fig. 2A (left) and the accompanying responses in Fig. 2B (left) and see also Supplementary figures for full scalp montage. There was no overall condition effect, nor any main effects or interactions for either analysis window when response amplitudes were compared in separate mixed model ANOVAs with a between-group factor for *context* (context 1, context 2) and a within-subject factor for *probability* (common, rare).

In adults and neonates, the absence of a differentiation in response to a rare and common sound with different physical properties is typically considered evidence that the two tones could not be discriminated^12,46–48^. Certainly, the brain responses of our two groups of infants show no significant evidence of a discrimination being made between the rare and common sounds in either control sequence. Thus, by these data alone, one would infer that these neonates were not able to discriminate the duration difference between 100 and 250 ms long tones. However, contexts 1 and 2 were quite clearly distinguished when the same tone sequences were concatenated alternatingly in a continuous sequence. Further, there was also indication of the rare properties being differentiated over time.

In the alternating contexts experiment (Experiment 3), another group of newborns were presented with the same tones as those used in the control contexts, but organized into an alternating sequence, switching back and forth between periods of context 1 and 2 modelled on sequences used in adults^41,49,50^ (see Fig. 1). The responses were remarkably different from those obtained in the control contexts. In Fig. 2A (right), B (right), the responses to tones presented in context 2 appear very similar to those in the control experiments, but the responses to tones in context 1 were clearly different. The morphological difference is evident in an earlier positive deflection in response to tones in context 1 of the alternating experiment: a positive shift begins approximately 100 ms earlier (that is, at about 50 ms in context 1 compared to ca. 150 ms in context 2). This morphological difference is not only evident between the context 1 and context 2 within the alternating experiment, but also between the control and the alternating context 1.

A repeated measures ANOVA of amplitudes obtained in the alternating sequence with within-subjects factors of *context* (context 1, context 2) and *probability* (common, rare) revealed a significantly larger positive polarity response over 50–150 ms in context 1 (*F*(1,27) = 6.269, *p* < 0.019, η^2^ = 0.188) reflecting this earlier positive response to tones in context 1 than 2. A mixed model ANOVA comparing context 1 early responses between the corresponding control and alternating experiments also indicated significantly more positive responses over 50–150 ms in the alternating sequence group than in the control group (*F*(1,64) = 16.968, *p* < 0.001, η^2^ = 0.210). There were no significant main effects or interactions for the equivalent comparisons of response amplitudes for context 2. These differences are clear in Fig. 2 where the mean amplitude for this period is clearly more positive in the alternating context 1. The same repeated measures ANOVA was applied to the responses over 150–300 ms revealing no significant differences nor interactions. In summary, unlike for the two control contexts, the infants responded differently to the same two contexts when they occurred within a single sequence of sound: the dominant positivity began earlier in alternating context 1, than in alternating context 2, with only context 1 differing from either control context. There was, however, no overall significant differentiation between the common and rare tones in either context, for either window, in either experiment.

**Figure 3:**
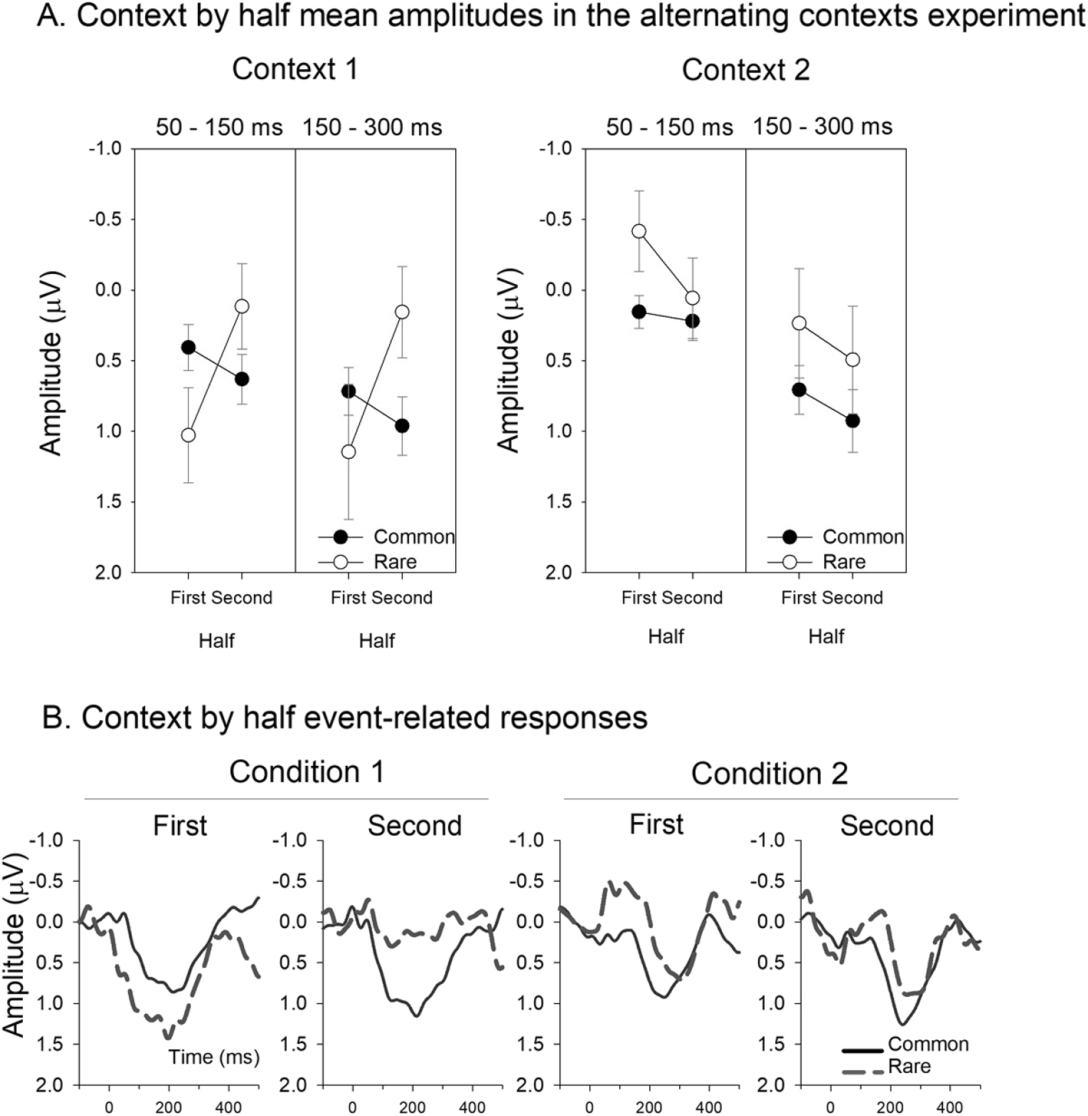
(**A**) Group mean amplitudes in the alternating context experiment over 50–150 ms and 150–300 ms from tone onset for the common and rare tone responses for context 1 (left) and context 2 (right) for the first and the second 2.1 min long periods within the context blocks. The error bars represent the standard error of the mean. (**B**) Group averaged event-related brain responses at Cz for the common and rare tones in context 1 (left) context 2 (right).

Given evidence that the infants could differentiate the contexts, and evidence in adults that the precision-weighting on auditory responses change dramatically over time within alternating sequences^41,49,50^, the data was further explored for evidence of probability differentiation by separately examining responses obtained from the first 2.1 min and second 2.1 min periods (1st and 2nd half, respectively). The component amplitudes for these divisions of the data are presented in Fig. 3A and the accompanying responses in Fig. 3B and see also Supplementary figures for full scalp montage. The data in Fig. 3, once again, display the much larger early positivity in response to context 1 tones in general, but there are also signs of differentiation between the rare and common tones in the second 2.1 min period over the later (150–300 ms) measurement window. A repeated measures ANOVA [*probability* (common, rare) × *context* (context 1, context 2)] was conducted to test the responses for effects of probability difference, separately for the first and the second 2.1 min periods and for the early and late measurement intervals. Examination of the responses in the first 2.1 min period revealed a significant effect of context over the 50–150 ms time window (*F*(1,27) = 11.393, *p* < 0.002, η^2^ = 0.297), where responses were significantly more positive in the first than the second context. The effect was further modified by tone probability (*F*(1,27) 5.464, *p* < 0.027, η^2^ = 0.168) with the differences being larger between rare than between common tones, of which only the former reached significance (*t*(27) = 4.034, *p*_holm_ < 0.001). There were no significant main effects or interactions for amplitudes measured in the 150–300 ms window over this first 2.1 min. However, importantly, no significant main effect was found for tone probability over this first 2.1 min period for either context.

In contrast, examination of responses in the second 2.1 min period revealed no differences over 50–150 ms. However, there was a main effect of probability for the 150–300 ms period (*F*(1,27) = 7.284, *p* < 0.012, η^2^ = 0.212). As can be seen in Fig. 3, the responses to the rare tones were significantly less positive (more negative) than those to the common tones (*m* = 0.325 µV vs. *m* = 0.944 µV, respectively). In summary, the differentiation in the event-related brain responses within the first 2.1 min primarily reflected the context that the tones were presented in, but by the second 2.1 min the tone probabilities were differentiated with the rare ones eliciting more negative response over the 150–300 ms; the same window as previously observed to capture a negative mismatch response^16^.

## Discussion

The context-dependence of neonate responses to sounds imply that the infant brain should be considered an intelligent consumer of information rather than as a passive processor of sound sequence material. Internal models of the environment are proposed to contain an estimate of how volatile the environment is (cf. “precision” in predictive coding theories^23,25^). The estimate of volatility/precision then weights the brain response leading to changes in response amplitude over time. Where a model has been formed based on highly reliable information, the response to a model-matching sound should be very suppressed and responses to model-mismatches highly salient^23,25,51^. The classic oddball sequences used for control contexts 1 and 2 are considered ideal learning environments for building precise internal models provided that the brain can distinguish between the common and rare elements. Earlier studies indeed suggest that the infant brain can distinguish the durations used here^15,16^, and the present data from the alternating sequences indicates that our infants did also discriminate the two durations. The key question is why the neonates did not do so in the classic oddball control sequences. The main findings of the present study are discussed below in relation to a thesis that the infant brain is sensitive and responsive to the information carried by sound, and that brain responses are adjusted to this determination accordingly. This sensitivity to the information carried by sound would be reliant upon the extraction of regularities in how features and objects co-occur in the environment over longer timescales which expands the view of statistical learning to the large scale structure.

The general morphology of the neonatal ERPs recorded in the current study conformed to the pattern observed in many previous studies: a negative waveform in the 50–150 ms time window followed by a dominant centrally positive response in the 150–400 ms time range. The two contexts used in these experiments were characterized by different tendencies towards two sounds that differed from each other in duration (100 ms and 250 ms). The responses elicited to the common and rare instances of these two sounds were remarkably similar in the control experiments. In contrast, the juxtaposition of the two contexts in the alternating sequence revealed an ability to discern the boundaries within the sequence where the probabilities of the two sounds changed, moving from a stronger tendency towards encountering the shorter tone after a period of hearing a stronger tendency towards encountering the longer tone and vice versa. The key indicator that the babies detected these boundaries lies in the point at which the positive shift onsets in the response morphology, which is much earlier for context 1 than 2, and much earlier in context 1 for the alternating experiment than for the control experiment. There was therefore a strong dependence of the morphology of the infant response based on the very long-term structure of the listening environment.

There are a number of properties that could influence the responses to tone changes at the boundaries through a process called adaptation (note, here we refer to adaptation in larger scale responses not at individual neuronal level, for distinctions^52,53^). Stimulus specific adaptation is an important mechanism through which the auditory system tunes responsivity to reflect redundancy and sharpen discrimination ability^54^ and distinguish information carrying signals from background noise^55^. The processes underlying how sound statistics affect adaptation are complex and sophisticated^52,53^, and while they are argued to be insufficient to fully account for deviance sensitivity^24^, they could contribute to the general context effect in the alternating sequence. Each of these is discussed in turn with respect to how well they fit the current data. Firstly, sounds of different duration carry different levels of sound energy^56^, and as such, it is possible that context 1 and 2 could have been differentiated based on a general shift in sound intensity over time. This sensory property-based distinction could be considered consistent with an effect appearing early within the event-related response^45^, possibly even earlier than the point of difference in tone length at 100 ms (see Fig. 2, alternating condition). The differences in sound duration used here can affect the degree of adaptation raising the possibility that context 1 responses are more positive in the early period because of a higher level of adaptation. However in adults, longer duration tones have been shown to increase the amplitude of the positive component that occurs at about 200 ms after tone onset, but not the earlier components^57^. Another feature of the contexts that could affect adaptation is the silent period between tones in the alternating sequence which was typically 400 ms in context 2 (100 ms common tones with 500 ms stimulus onset asynchrony (SOA)) but only 250 ms in context 1 (250 ms common tones with a 500 ms SOA). In adults, this difference in silent interval changes the degree of adaptation. However, once again in adults this is evident mostly approximately 200 ms after tone onset^57^. Finally, the silent intervals are slightly longer in the control conditions (550 ms for context 1 and 700 ms for context 2 which) which also could be associated with different levels of adaptation and could again affect the amplitude of the positive component that occurs at about 200 ms after tone onset. However, a difference in adaptation is not evident between the two control conditions. Further, this effect would create a more positive response in the control context 1 than in the alternating context 1^57^. In contrast the opposite of this has been found here. In sum, the current data does not support the notion that adaptation alone is likely to explain the effects in neonates distinguishing the different contexts or the alternating and the control sequences. Therefore, instead it is proposed that the distinction in the responses only emerges where the statistical properties of sound carry information about a longer-term structure within the sound sequence. In other words, we propose that the babies can distinguish between the two sound contexts, but they do so only when differentiation is meaningful to isolate information needed to encode larger scale structure.

It was also only when the sounds were embedded within a longer structure that evidence of an ability to discern one sound as a local rare outlier emerged. When responses from the first and second half of the contexts were separately averaged, it became evident that responses appearing initially within a “new” context tended to reflect the differentiation of the context only (i.e., the difference in early positivity). Later on, when the context had been stable for a while, a significant longer-latency negative shift was seen in response to the rare tone in context 1, consistent with prior observations of deviance detection^16^. The infants were detecting the sound that was out-of-place or unexpected in that context and responding in a similar way to that which we see in adults (i.e., more negative response^47,58,59^). Interestingly, the data also suggest this was particular to the first context heard. The first context, regardless of whether long or short tones are common, is also associated with higher precision models in adults^41–44^. The alternating sequence responses therefore provide evidence that newborns can not only determine the sequence structure^60^, but can also form internal models that build up in precision over time enabling an outlier to be detected. A progressive refinement of cortical response in neonates over time has also been seen in statistical learning of language^8^ which is also compatible with the concept of precision-weighting but in the present case it is demonstrated with respect to relative probability. These observations add to a body of work showing how such learning is shaped by many longer timescale characteristics including hierarchy, order, entropy, and uncertainty^9^.

So why were infants not sensitive to the outliers when presented within a listening context that should have been ideal to observe differential responses? One possible reason for the longer-term structure of the sound sequence influencing sound differentiation may relate to a trade-off between the information value of maintaining precise internal models and the associated investment of effort. In the control sequences, the babies heard 3000 tones in a constant uninterrupted probability arrangement over a 40-min period. In the alternating sequence, the exposure to the two contexts occurred in blocks of 500 stimuli: thus, the maximum period of exposure to one sound probability arrangement was shorter than 7 min. Similarly, in prior studies showing the ability of young infants to distinguish between sounds of two different durations^15,16^ the infants always heard sequences of less than 7 min duration with breaks between successive presentations. Thus, a potential explanation for the absence of tone differentiation in the current control sequences is that rare changes to the duration of sound within a long constant sequence carried insufficient information to warrant considering a different model, as there was no alternative offered by the sound input. Once again, the babies can distinguish between the two sounds, but perhaps cease to do so when it offers no new information about structure.

When exposed to oddball sequences over a long period of time, a broader and more inclusive model that includes both sound durations would avoid attention capture by the unimportant deviations, potentially conserving energy for modelling other aspects of the environment^23,25,61^. Under this broader more inclusive model, all sounds heard may be captured as predicted such that the absence of differentiation in the responses does not reflect an inability to distinguish them, but rather a determination that distinguishing between them carries insufficient benefit to warrant the effort involved. In contrast, when these same two sounds occur within the alternating sequences, two different acoustic states are present in the environment and modelling these two different states may become energetically more beneficial. This suggests that the optimization of resources, a potential driver of cognitive development^62^, is already evident in the first few days after birth. The above account is fully compatible with a predictive coding theory of brain function suggesting that a fundamental operating principle is to seek to reduce variational free energy, that is to reduce surprise by utilizing statistical information to anticipate upcoming states^23,61,63^. Observations enable accumulation of a probability density over their hidden causes, and through a process of active inference, an internal model of the most likely next state can be inferred and refined over time based on the adequacy of the fit to new evidence. The optimization of a model to reduce free energy can be governed by weighting whether to aim for accuracy of predictions over the shorter or longer term by the energy required to achieve that level of accuracy. Developing or maintaining sensitivity to the two sound durations is beneficial to extracting the predictable structure in the alternating sequence, but not in the control sequence. This kind of contextualized processing is another example of how expectations can shape responses to sound^64^, with the present observations suggesting that some such modulations are so fundamental to learning that evidence can be seen in the first few days of life, even in sleeping neonates.

A limitation of the present study, as noted in the introduction, is that the stable componentry of adulthood only emerges during childhood. The responses observed in neonates are incomparable to those in adults (e.g., neither the N1 nor N2 component has an equivalent in neonates; unlike the amplitude of the adult response, MMR is affected by acoustic parameters of the deviant stimulus, etc.^32^) which complicates the study the computational principles underlying them (see for discussion^32^). There are a number of studies, for example, that refer to a larger central positivity as being indicative of deviance detection for some features in infant data^33^ but this was not observed here and may not be how duration deviance is reflected in neonate ERPs where previously, a more negative response has been observed^15,16,65,66^. Notwithstanding, we suggest the context-sensitivity and changes over time in responsiveness present in these neonate data are indicative of the way perception is shaped by modelling of complex patterning in the environment.

In sum, the infant data presented here provide two insights into the newborn’s readiness to process sounds within context. Perhaps not so surprisingly, the newborn is a discerning listener. Far from being a passive receptacle for sensory experiences, the neonate brain appears to be governed by determinations of a trade-off between information value and energy efficiency. This cost–benefit equation seems to result in a conservation of energy in situations, where no alternative models can be considered. However, the neonates do adopt more precise models when a single model cannot adequately account for multiple states. Under these circumstances there is clear evidence of altered responsiveness that enables a gross differentiation of states (between the two contexts) and the emergence of differentiation of probable features in a precision-weighted manner. These feats are important for infants to learn from the environment, finding regular sequences of events within constantly changing stimulation of typically several sources of information/stimulation present concurrently. This ability would seem pivotal to explain the readiness with which infants learn critical interaction skills such as understanding speech and entering into communication exchange and dialogues^60,67^.

## Materials and methods

### Participants

Electroencephalographic (EEG) data useful for the current analyses was recorded from 110 (56 male) healthy, full-term infants 0–6 days postpartum. Data collected from another 14 infants had to be discarded: three due to recording errors (no trigger was recorded) and eleven due to failing to meet criteria for data quality (see the “EEG recording and analysis” section below). The infants were divided into three groups (experiments): the control context 1 group (n = 38, 20 male) had a mean gestational age of 39.19 weeks (SD = 0.84), birth weight 3472 g (SD = 522.21); the control context 2 group (n = 41, 24 male) had a mean gestational age of 39.48 weeks (SD = 1.03), birth weight 3421 g (SD = 416.95) and the alternating contexts group (n = 28, 12 male) had a mean gestational age of 39.12 weeks (SD = 1.03), birth weight 3404 g (SD = 457.50). These sample size are commensurate or in excess of those in prior similar studies^15,16^ and g*power estimates suggest the power of observing a significant mismatch response if present exceeded 0.99. All participants had an Apgar score of 9/10. Infants were predominantly asleep during the recordings, in quiet sleep 78% of the time, and in active sleep 16% of the time. Informed consent was obtained from one (mother) or both parents. The experiment was carried out in a dedicated experimental room. Experiments 1 & 2 were carried out concurrently with babies allocated to experiments in counterbalanced order. Experiment 3 was carried out separately with all babies hearing the same sequences. Importantly, all recordings were carried out in the same manner, in the same setting. The study was conducted in full accordance with the Declaration of Helsinki and all applicable national laws and was approved by the relevant ethics committees: Medical Research Council-Committee of Scientific and Research Ethics (ETT-TUKEB), Hungary.

### Sound sequences

Each group were presented with a sequence of harmonic tones delivered binaurally using the E-Prime stimulus presentation software (Psychology Software Tools, Inc., Pittsburgh, PA, USA) with ER-1 headphones (Etymotic Research Inc., Elk Grove Village, IL, USA) connected via sound tubes to self-adhesive ear cups (Sanibel Supply, Middelfart, Denmark) placed over the infants’ ears.

The sequence presented in the control conditions were based on sounds used previously in similar infant studies yielding significantly different responses to common and rare sounds (11, 12). The two harmonic complex tones of different duration (100 ms, 250 ms with 5 ms cosine rise and fall each), comprised of three pure sine frequency components (500 Hz, 1000 Hz and 1500 Hz with − 5 dB/step intensity). All tones were delivered with a uniform 70 dB intensity. The tones were presented in a typical oddball design^11^ with one sound being common (p = 0.85) and the other rare (p = 0.15), and the sounds occurring pseudo randomly constrained such that a minimum of three common sounds occurred between rare sounds. In context 1, the long sound was common, and the short sound was rare, and in context 2, the probabilities were reversed.

A diagram depicting the structure of the sequences presented to each group is presented in Fig. 1. In experiments 1 and 2 (control) 3000 tones were presented in a single unbroken sequence at a regular 800 ms stimulus onset asynchrony (SOA) with one group receiving a sequence made up as context 1 and the other as context 2. In experiment 3 (alternating contexts), the tone sequences comprised the same two sounds of different duration but an SOA of 500 ms, which were presented in groups of 500 alternating back and forth between context 1 and 2. The shorter SOA was necessary to preserve timing similarity to alternating sequences in adults^41^ and SOAs of 300–500 ms have been used successfully in other neonates studies^68,69^. Each group of 500 sounds began with a minimum of four occurrences of the new common sound. The sequence was presented three times with a 1 min silent period between them and always started with a context 1 block.

### EEG recording and analysis

EEG was recorded with Ag/AgCl electrodes attached to the Fp1, Fp2, Fz, F3, F4, F7, F8, T3, T4, Cz, C3, C4, Pz, P3, P4 locations according to the International 10–20 system. The reference electrode was placed on the tip of the nose and the ground electrode on the forehead. Data was recorded using a direct-coupled amplifier (V-Amp, Brain Products, Munich, Germany) at 24-bit resolution and a sampling rate of 1000 Hz. Data processing was conducted using the ERPLab^70^ plugin to EEGLAB^71^ within MATLAB. The EEG signals were passed through a digital bandpass filter between 1 and 25 Hz and epochs of − 100 to 500 ms with respect to the sound onset were extracted for each stimulus. All amplitude measurements and illustrations are referred to the 100-ms pre-stimulus interval, the baseline.

Epochs were subjected to an artifact rejection process where epochs with a voltage change exceeding 150 μV were rejected from analysis. The data quality was considered acceptable if a minimum of 50% of the epochs were retained for every condition tested, for electrodes F3, Fz, F4, C3, Cz, C4, P3, Pz, P4. Responses were averaged separately according to sound duration and probability (context 1: long common and short rare tone; context 2: short common and long rare tone). For testing the long vs. short discrimination, the responses in the alternating condition were further divided into averages that separately captured the first and second halves (the first and second 2.1 min period, respectively; see Fig. 3) within each context block. The separation of the first and second half of the context blocks has been very useful in analyzing data from adults as the responses change significantly over time^49,72^.

Two time windows were used to quantify the amplitudes within the central (Cz) responses, the electrode selected as it typically best captures deviance-related effects in neonates (e.g., ^32,34^). The first was the mean amplitude between 50 and 150 ms after sound onset to capture early components of the response. The second was the mean amplitude between 150 and 300 ms after sound onset which captures the period over which a significant difference was present in the response to common and rare tones in prior work (12) indicating that a mismatch to the prevailing internal model was detected. Separate ANOVA analyses were conducted on the 50–150 ms and 150–300 ms amplitude measurements. These are described in the results section with some being within-subject and others between-subject as required. Post-hoc Holm-Bonferroni corrected tests were employed to explore significant interactions. Effect sizes are reported as partial eta squared and the significance criterion level was α < 0.05. All statistics were computed using JASP (version 0.13.1^73^).

## Supplementary Information

Supplementary Information.
