# Supplementary material for: Relevance to the higher order structure may govern auditory statistical learning in neonates
Source: Sci Rep. 2022 Apr 7;12:5905. doi: 10.1038/s41598-022-09994-0 (PMC8989996; doi:10.1038/s41598-022-09994-0)
Supplement: Supplementary file 1 — Supplementary Information. [file 41598_2022_9994_MOESM1_ESM.docx]

Supplementary Figures

Supplementary Figures (S1-4) feature the full nine electrode montage presentation of the ERP data for context 1 and context 2 in the two control experiments and the alternating experiment. The purpose of presenting these figures is to offer interested readers the opportunity to observe more of a topographical appreciation of the data analysed from the Cz site alone in the main manuscript.


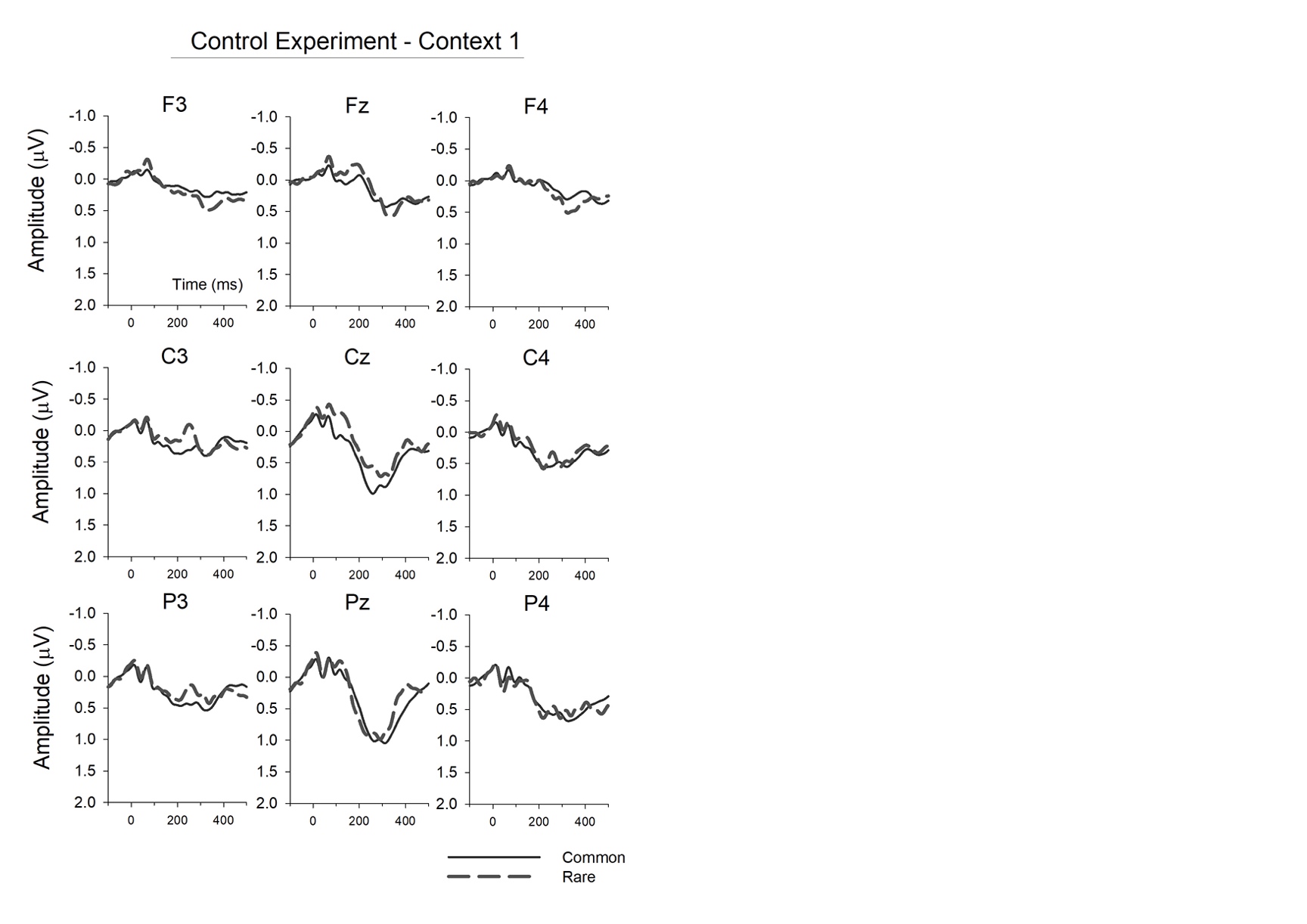


Figure S1. The group averaged ERPs to the common and rare tones presented within Experiment 1 in which the longer 250 ms sound was the common tone and the shorter 100 ms sound was the rare tone (i.e, context 1).


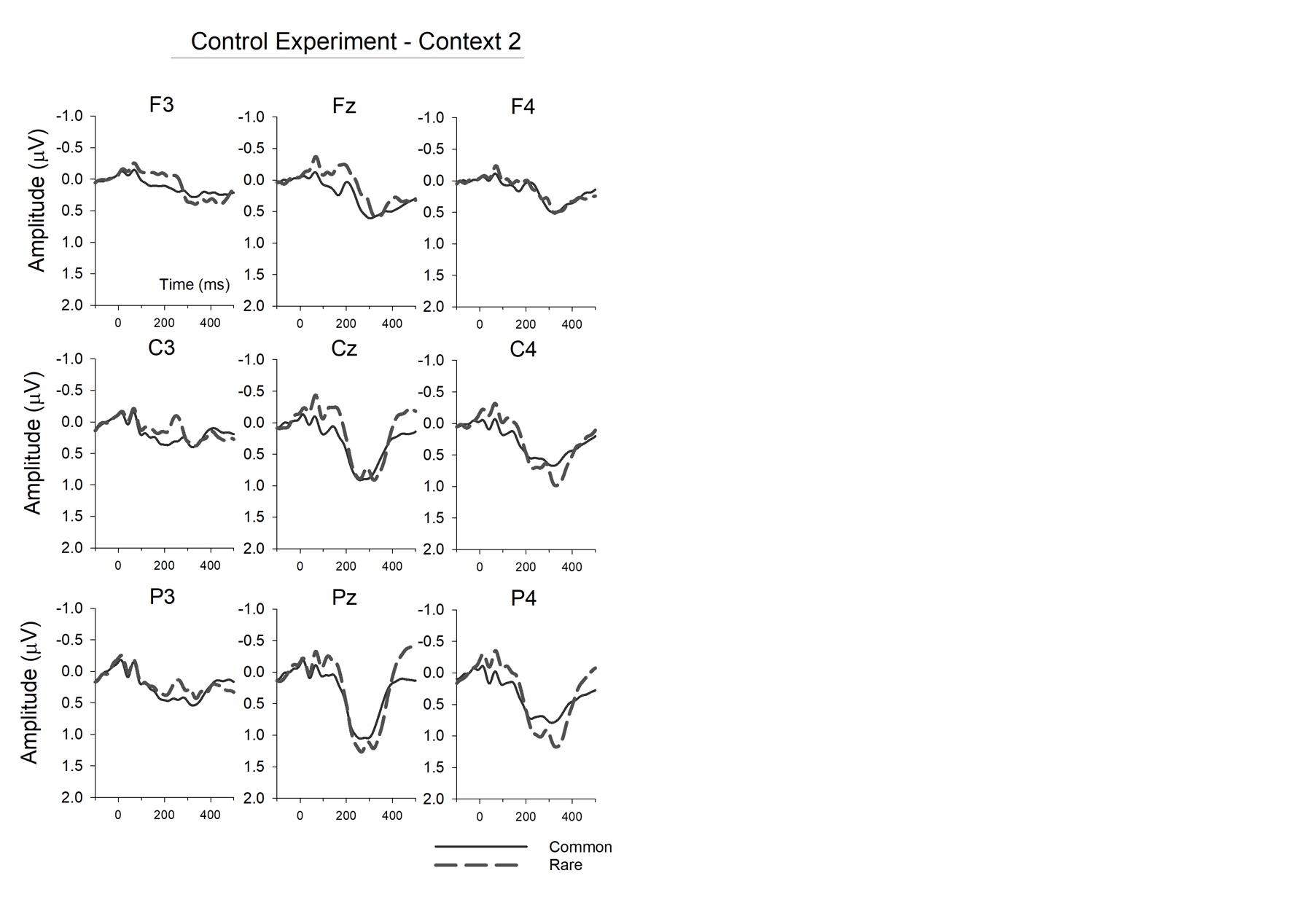


Figure S2. The group averaged ERPs to the common and rare tones presented within Experiment 2 in which the shorter 100 ms sound was the common tone and the longer 250 ms sound was the rare tone (i.e, context 2).


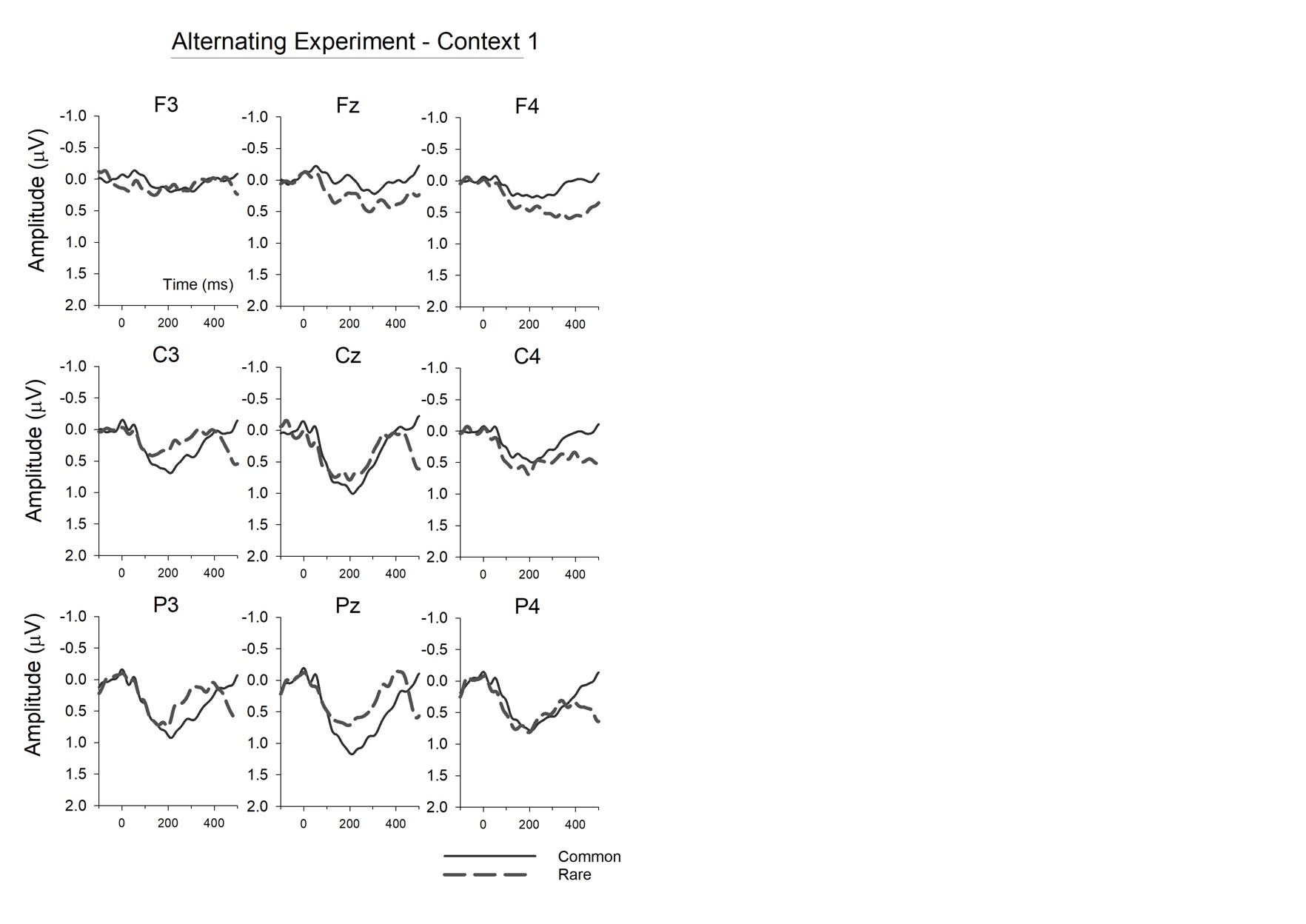


Figure S3. The group averaged ERPs to the common and rare tones presented within Experiment 3 for context 1 blocks in which the longer 250 ms sound was the common tone and the shorter 100 ms sound was the rare tone.


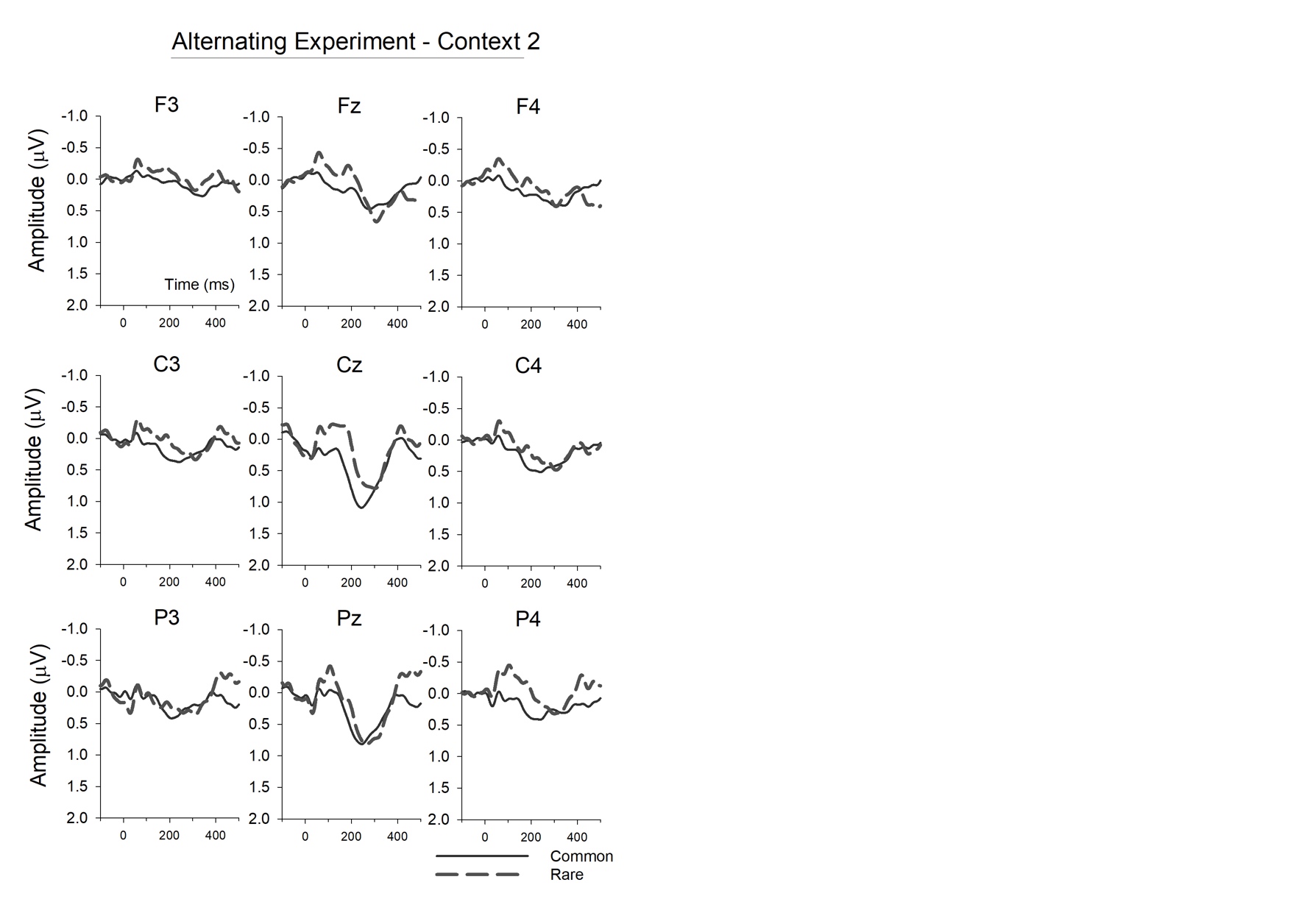


Figure S4. The group averaged ERPs to the common and rare tones presented within Experiment 3 for context 2 in which the longer 100 ms sound was the common tone and the shorter 250 ms sound was the rare tone.
